# An Accessible Method for Implementing Hierarchical Models with Spatio-Temporal Abundance Data

**DOI:** 10.1371/journal.pone.0049395

**Published:** 2012-11-16

**Authors:** Beth E. Ross, Mevin B. Hooten, David N. Koons

**Affiliations:** 1 Department of Wildland Resources, Utah State University, Logan, Utah, United States of America; 2 U. S. Geological Survey, Colorado Cooperative Fish and Wildlife Research Unit, Fort Collins, Colorado, United States of America; 3 Department of Fish, Wildlife, & Conservation Biology, Colorado State University, Fort Collins, Colorado, United States of America; 4 Department of Statistics, Colorado State University, Fort Collins, Colorado, United States of America; 5 Department of Wildland Resources and the Ecology Center, Utah State University, Logan, Utah, United States of America; University of Otago, New Zealand

## Abstract

A common goal in ecology and wildlife management is to determine the causes of variation in population dynamics over long periods of time and across large spatial scales. Many assumptions must nevertheless be overcome to make appropriate inference about spatio-temporal variation in population dynamics, such as autocorrelation among data points, excess zeros, and observation error in count data. To address these issues, many scientists and statisticians have recommended the use of Bayesian hierarchical models. Unfortunately, hierarchical statistical models remain somewhat difficult to use because of the necessary quantitative background needed to implement them, or because of the computational demands of using Markov Chain Monte Carlo algorithms to estimate parameters. Fortunately, new tools have recently been developed that make it more feasible for wildlife biologists to fit sophisticated hierarchical Bayesian models (*i.e.*, Integrated Nested Laplace Approximation, ‘INLA’). We present a case study using two important game species in North America, the lesser and greater scaup, to demonstrate how INLA can be used to estimate the parameters in a hierarchical model that decouples observation error from process variation, and accounts for unknown sources of excess zeros as well as spatial and temporal dependence in the data. Ultimately, our goal was to make unbiased inference about spatial variation in population trends over time.

## Introduction

Monitoring and detecting changes in population abundance, and determining why changes vary over space and time, are concepts that are central to ecology, conservation, and management [1], [2]. Obtaining accurate estimates of trends and other changes in population abundance is more difficult than it might appear given complicating issues such as density-dependence and stochasticity. Various sources of uncertainty in data and underlying ecological processes (*e.g.*, temporal or spatial autocorrelation and observation error) can confound inference about changes in population abundance [3].

Statistical models that have been used to assess changes in population abundance have ranged in complexity from linear regression to generalized linear models (*e.g.*, Poisson regression), generalized additive models, and time-series models [4], [5]. While increasing complexity in these models helps meet assumptions, none can simultaneously decouple process error from observation error, and account for both temporal and spatial autocorrelation. Hierarchical models may present the best statistical approach for assessing changes in population abundance across large spatial areas [6]–[8]. Hierarchical models are ideal for handling observational data because they allow for the explicit separation of observation and process error [6], [9], [10]. This is a key distinction, as hierarchical models do not require the assumption that either the data are collected without error, or that the processes underlying the data are known without error [3], [11], [12]. Ignoring observation error can lead to inaccurate estimation of focal parameters, such as vital rates and trends in abundance [3], [13]. In addition, ignoring uncertainty in ecological processes themselves can result in spurious conclusions [10]. For example, if spatial autocorrelation is present in the data, but not accounted for, the variance associated with the parameter will be estimated to be smaller than it should be, potentially causing the researcher to conclude that a result is statistically significant when, in reality, it is not [14].

While extremely useful for ecological applications, a drawback of the hierarchical modeling approach is the required background in statistics and computer programming that is necessary to implement sophisticated models incorporating spatio-temporal dynamics. Markov Chain Monte Carlo (MCMC) is a powerful method used to sample from the hierarchical Bayesian posterior distributions of parameters, or determine Maximum Likelihood Estimates via data cloning [15]. Unfortunately, MCMC involves high computational time and the challenge of correctly interpreting output (*e.g.*, convergence diagnostics). Estimation of state variables in hierarchical models with the Kalman Filter, a set of recursive equations, is also limited by modeling assumptions [6]. Recently, a statistical algorithm has been developed for obtaining posterior distributions from Bayesian hierarchical models that does not involve the use of MCMC. Based on Laplace’s work with integral transforms [16], this new method is referred to as integrated nested Laplace approximation (INLA), which uses a three-step process involving Laplace approximations and numerical integration to derive posterior distributions for the parameters of interest [17]. Although INLA is only capable of handling hierarchical specifications with generalized linear process models and a latent Gaussian random field, it has the added advantage of being more accurate than MCMC within reasonable computation times [17]. Additionally, the development of a readily available R package [18] makes INLA accessible to users with a reasonable background in parametric statistics [17].

Hierarchical models have been used to assess trends in abundance while accounting for process error and observation error [19], incorporate mechanisms of density-dependence [6], [12], [20], and estimate second-order spatial autocorrelation to obtain unbiased estimates of precision in population dynamics across a landscape [8]. However, fully spatio-temporal models of population dynamics can still be difficult to visualize and implement [21]. This is not altogether surprising, given the statistical and computational requirements that have, until now, been needed to estimate parameters in sophisticated hierarchical models that account for first- and second-order autocorrelation processes across both space and time.

Fortunately, INLA methodology and the associated R package [18] developed by Rue et al. [17] make the implementation of hierarchical spatio-temporal models relatively easy. This package allows for researchers to easily incorporate various structures into the process model that are relevant to modeling population abundance. Latent models such as seasonal variations [22], spatial effects [23], and autoregressive models (*i.e.*, AR(1) [6]) can be specified using INLA. Additionally, these process models can be compared using deviance information criterion (DIC [24]) in INLA, thus allowing the researcher to determine which models best describe the given population. The same comparison using DIC can be done when selecting among error models for the data. In addition to latent (or unobservable) variables, covariates of interest and temporal trends can be specified directly in the process model to account for additional sources of variation in the process. The ability to account for first- and second-order processes in turn allows the researcher to make inference pertaining to the covariates of interest while also learning about the latent variables.

In this paper, we utilize a long-term, continental-scale dataset of abundance counts of the lesser and greater scaup (*Aythya affinis* and *A. marila*, respectively) to illustrate how INLA can be used to fit models with underlying spatio-temporal mechanisms and autocorrelation to better understand the processes governing population dynamics. We then discuss how additional processes can easily be incorporated into our case study to address an array of ecological questions.

### Case Study

Every year, the U.S. Fish and Wildlife Service and Canadian Wildlife Service conduct the North American May Breeding Pair Survey (BPS), which provides a rich source of demographic data on 10 focal duck species (as well as others). The BPS includes areas in the north-central United States, much of western Canada, and Alaska; purposefully covering a large portion of each species’ breeding range [25]. This survey has been conducted every May through June since 1955 using aerial transects [26]. Surveys are flown at approximately 193 kilometers per hour at an altitude of 27–30 meters. Within each stratum, the largest sampling unit of the survey, pilots fly multiple transects, each comprised of 28.8 km strip-segments. The number of segments sampled in a transect ranges from 1 to 35, and has changed over time. Strata units vary in size, and are based on geographical and political boundaries. Observers count duck species, and whether or not the ducks are paired (with a mate), single drakes, or in mixed-sex groups.

Two particular species of interest that are surveyed during the BPS are the lesser and greater scaup, which are counted collectively as ‘scaup’ due to their similar appearance. Scaup are the most abundant and widespread diving duck in North America, and are important game species [27]. Since 1978, however, the continental population of scaup has declined to levels that are 16% below the 1955–2010 average and 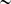
34% below the North American Waterfowl Management Plan goal [25]. This decline has sparked concern amongst hunters, management agencies, and conservation groups alike [28]. The greatest decline in abundance of scaup appears to be occurring in the western boreal forest, where populations may have depressed rates of reproductive success, survival, or both [28]–[31]. However, the specific vital-rate pathways responsible for the decline are not known [32]; nor is there a consenus on the underlying mechanisms that may have caused the population decline. Leading hypotheses include: decreased food availablilty during spring migration, and consequent arrival on the breeding grounds in poor body condition that could inhibit reproductive success (*i.e.*, the Spring Condition Hypothesis [28], [33]–[35]); and toxins (particularly selenium [36], [37]) acquired on the Great Lakes and other migratory stopover locations that could inhibit both reproduction and survival [38]. However, DeVink et al. [39] and others [36], [37] concluded that selenium and mercury levels are low in boreal scaup, and not likely responsible for the population decline in this important breeding region. Moreover, toxins do not seem to be inhibiting scaup vital rates [40]. Studies examining the Spring Condition Hypothesis have produced conflicting results across spatial regions [39]. Other hypothesized drivers include anthropogenic development of the boreal forest (*e.g.*, hydropower dams, logging, oil and gas extraction, and mining) and climate change. Recently, Drever et al. [41] found that decreasing snow pack on the breeding grounds in the boreal forest (and thus subsequent pond conditions) is negatively correlated with regional population growth rates in scaup.

To better understand the causes of the decline, a high level of importance has been placed on retrospective analyses that accurately identify the spatial and temporal changes in population abundance [42]. A spatio-temporal analysis of population trends would thus help identify the regions where scaup abundance has declined most severely, where no change has occurred over the long-term, and even identify areas where abundance may be increasing. Such information is useful for directing management and conservation actions toward the most important areas, and would allow researchers to gain clearer insight into the mechanisms that might be causing declines in some regions and increases in others.

## Hierarchical Model

To account for process and observation error, we used Bayesian hierarchical models to examine change in scaup abundance for each stratum between 1957 and 2009 (scaup data were scarce in 1955–1956). Here, our focus is on the delineation of scaup recorded in breeding pairs, rather than total scaup abundance, as these pairs best represent the breeding potential of the population. We did not utilize data regarding single drakes due to the skewed sex ratio in scaup [28]. Further, counting methods for the mixed-sex non-breeding groups changed in 1975 [26]; thus, use of these data would confound long-term analysis from 1957 onward. Our basal unit of data was the number of pairs, 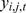, observed on each segment *i*, in stratum *j*, in year *t*. The hierarchical approach allowed us to incorporate spatial autocorrelation among strata into the process model while also gaining insight into the changes within each stratum. We also incorporated terms in our process model to account for temporal autocorrelation in the data, which often occurs in temporally dynamic systems. We also selected between data models to determine which models best fit the data. Bayesian hierarchical models are usually comprised of three ‘submodels’: a data model describing the distribution of the data, a process model specifying the underlying mechanisms that give rise to the data, and the parameter model indicating the distributions of the parameters in the process model [43]. Below, we outline each of these model components for our case study.

### Data Model

Based on preliminary analysis, the BPS scaup data appeared to be overdispersed, containing a disproportionately high number of zeros along with a high variance relative to the mean [44], [45]. Thus, we considered two potential data models, a negative binomial model where 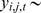
NegBinom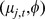, and a zero-inflated negative binomial model where
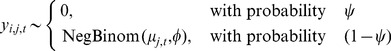
(1)where 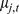 denotes the average abundance of counted pairs for segments *i* = 1,…,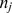 in stratum *j* = 1,…,*m* during observation period *t* = 1,…,*T* (*i.e.*, years 1957–2009). Models were then compared using DIC. We considered the zero-inflated model because it accounts for excess zeros, which can arise from more zero counts in a dataset than would be well described by a typical data model, and can occur from either ‘false’ or ‘true’ zeros. False zeros may arise due to birds that were present during the survey, but unobserved, or not present during the survey (*e.g.*, temporary emigration to a loafing pond). Unlike these false zeros, true zeros could occur when scaup do not fully occupy their suitable habitat, or when the habitat is simply not suitable [46]. One of the benefits of hierarchical modeling is that one can specify a distribution that properly supports the data (*i.e.*, the range of values *y* can take on), alleviating the need for direct data transformations (*e.g.*, log or arcsine) that are commonly used to specify linear process models. This is a valuable property of generalized linear models, given that models based on negative binomial distributions can perform better at modeling count data than transformed data with Gaussian model assumptions [47].

### Process Model

We incorporated each data model into a simple log-linear regression model with hierarchical terms (*i.e.*, random effects) that help account for spatial and temporal autocorrelation. This allowed us to analyze the temporal trend for each stratum separately, as well as determine the relative influence of both the fixed (the 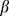 parameters) and random effects (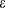 and 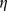) on relative scaup abundance. Using 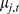 from the data model (1), the process model was specified as
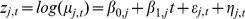
(2)where 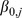 parameters are stratum-specific intercepts, while the 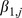 are trend parameters for each stratum that together model first-order spatial and temporal variation in abundance. Spatially and temporally correlated errors are represented by 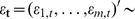
N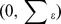, and 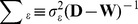 for all time *t* = 1,…,*T* representing spatially correlated errors and 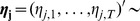
N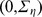 for all strata *j* = 1,…,*m* representing temporally correlated errors. The 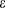 and 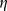 terms incorporated into the model (2) are intended to account for variation in both the spatial and temporal dimensions of the system, respectively, 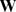 is a proximity matrix describing the neighborhood structure, and 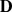 is a diagonal matrix with the row sums of 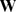 as diagonal elements [48]. Again, if these sources of uncertainty are not accounted for, erroneous inference could be made because model assumptions would not be met [49].

Rather than standardize the survey area to some sort of grid as past studies have done (*e.g.*, Gardner et al. [50]), we instead used the existing spatial structure present in the survey, and based our spatial field on the strata units as areal regions. This provided the basis for a conditional autoregressive structure (CAR [51], [52]), while also yielding results that were directly useful for management of the species. The CAR model is a commonly used spatial model for areal processes, and incorporates dependence among areal locations through the neighborhood structure of the strata. In constructing the proximity matrix, **W**, we calculated the Euclidean distance between the center of each stratum, and then denoted all pairs of strata as neighbors if they were within a threshold distance of 7.75 decimal degrees of each other. This distance was chosen for this case study because it was the shortest distance at which all strata had at least one neighbor, and constructing the proximity matrix in such a way accounted for the more spatially isolated strata in the north and the highly connected strata in the south. Thus, in our case, **W** was a 52 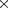 52 matrix (dimensions determined by the number of strata) with elements 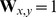 indicating stratum 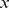 and stratum 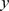 were neighbors, and 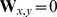 if otherwise.

In addition to spatial structure, we also accounted for latent temporal dependence in the system. We let 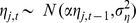, where 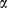 is the autocorrelation parameter, which introduces a temporal correlation structure on 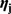, the vectors of residuals about the modeled log counts in stratum *j* over time, such that 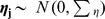. Given these parameterizations, the entire latent (*i.e.*, random-effect) process is a Gaussian Markov random field, which allows for the use of INLA to approximate posterior distributions. Also, in a biologically meaningful context, the model for 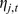 implies latent Gompertz growth, since the population at time *t* is dependent on the population at time *t*-1 on the log scale.

### Parameter Model

The parameter model consists of all the prior distributions assigned to the unknown parameters in the process model. The overdispersion parameter for the negative binomial distribution was specified as 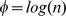, where *n* is the original negative binomial size parameter, and then modeled as 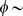 N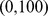. The regression parameters were specified with conjugate Gaussian priors so that 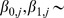
N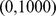, for *j* = 1,…,*m*. The variance component was specified in terms of precision [17], and was given a conjugate gamma prior, 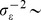
Gamma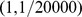. Lastly, given the reparameterization of 
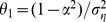
 and 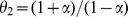, 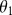 was assigned the prior 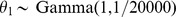 and 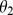 was assigned the prior 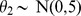. These reparameterizations are suggested for implementation of INLA for ease of processing [17].

### Model Implementation Using INLA

Combining the data, process, and parameter distributions to form a hierarchical model yielded the posterior distribution:
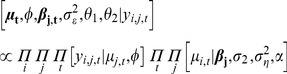
(3)

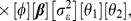
where we use square brackets ‘
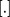
’ to denote a probability distribution. Typically, the above model would be fit using MCMC, usually using a combined Gibbs sampler and Metropolis-Hastings algorithm after solving for the full-conditional distributions where closed-form solutions exist [48]. Instead, we used INLA to approximate the marginal posterior distributions of the parameters of interest [17].

Many models used in ecological applications are based on latent Gaussian random fields, ranging from linear regression models, temporal models, or similar spatial and spatio-temporal models. By making use of these latent Gaussian models, INLA is capable of approximating the posterior distribution with high accuracy at a much faster computational rate than MCMC [17]. Unlike MCMC, INLA is not an iterative stochastic procedure, but rather a multi-step mathematical process used to approximate posterior distributions. According to Rue et al. [17], INLA performs a sequence of approximations (*i.e.*, first the posterior marginal of the parameter of interest, then the posterior) and then combines them using numerical integration. Additionally, INLA cannot approximate posterior distributions of nonlinear transformations of process model parameters, which may be a drawback for some ecological studies, but does not interfere with research like that presented in our example. We implemented INLA using the R package [18] called ‘INLA’ [17], and provide annotated partial code pertaining to our example in the supporting information (Text S1).

## Results

When comparing a negative binomial with the zero-inflated negative binomial (ZINB) data model, the ZINB had the lowest DIC (24,521 compared to 24,933 for the negative binomial). Additionally, we compared models with no random effects, only a spatial random effect, only a temporal random effect, and both random effects. Of these, the model with only a temporal random effect was the best (DIC of 24,427). Although this model was ranked best by DIC, we chose to include the parameter for the spatial random effect in order to avoid erroneous conclusions about estimates of the fixed parameters (DIC of 24,521).

**Figure 1:**
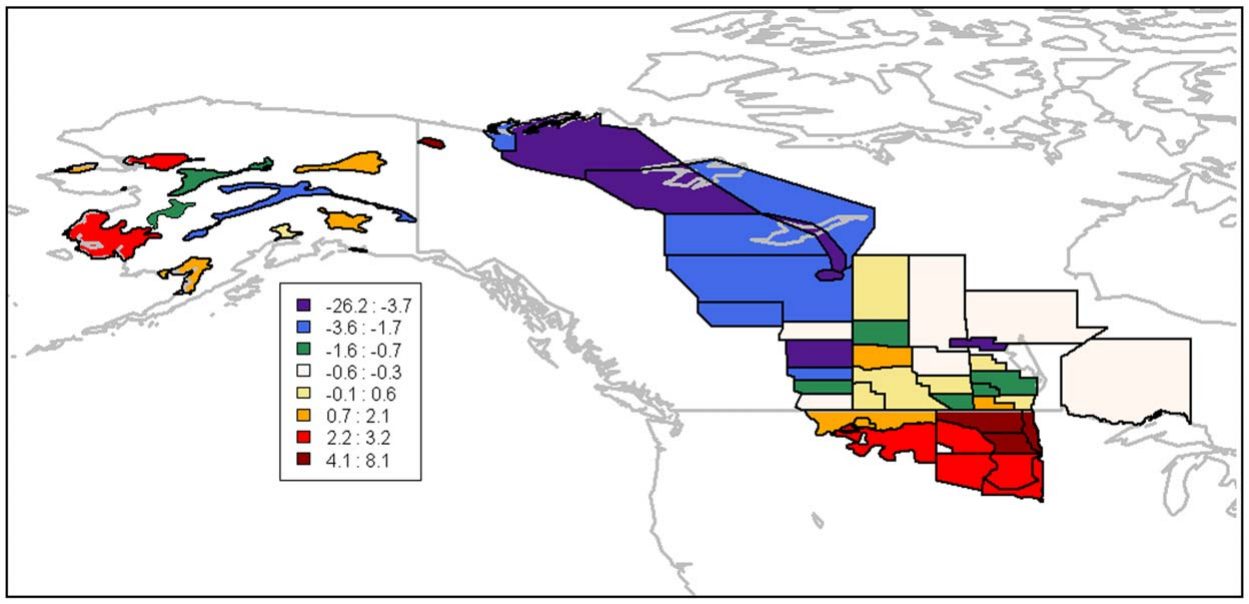
Changes in counted breeding pair abundance since 1957 based on posterior distributions for the 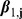 coefficients. Negative numbers indicate pairs lost and positive numbers indicate pairs gained. Note that not all differences are estimated to be statistically different from zero.

**Figure 2:**
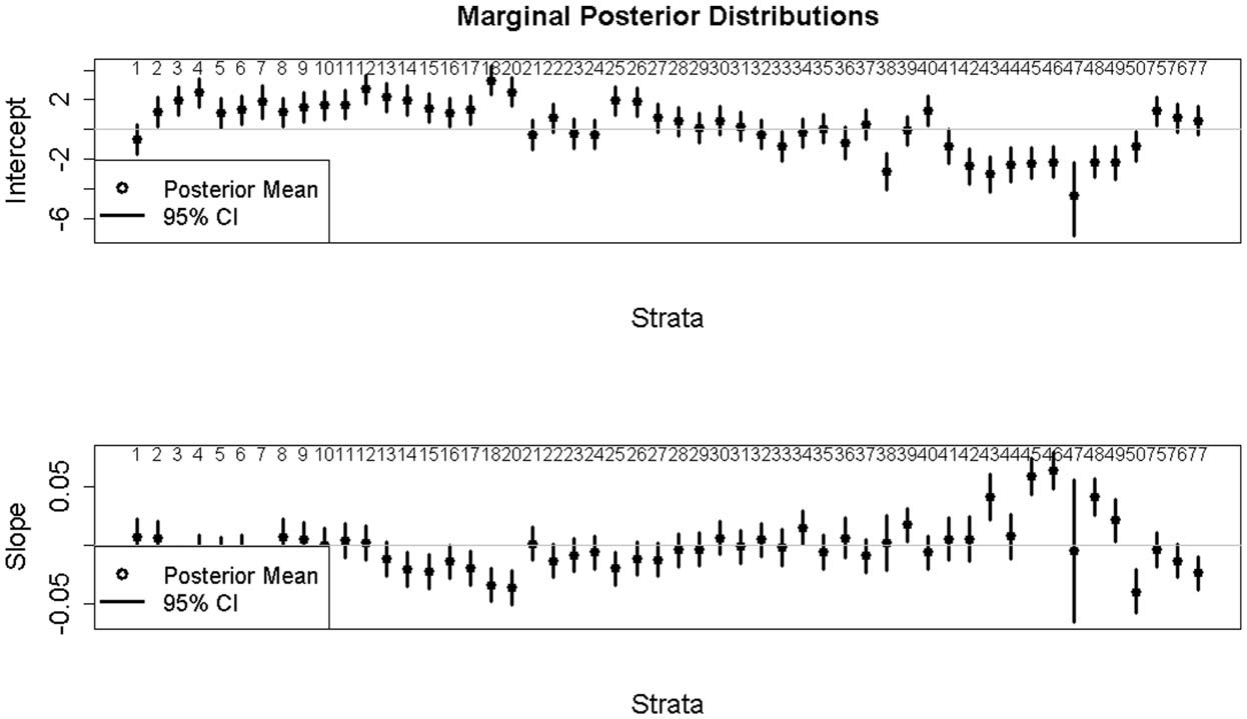
Marginal posterior distributions for the 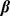 coefficients controlling for general trend using the zero-inflated negative binomial data model.

**Figure 3:**
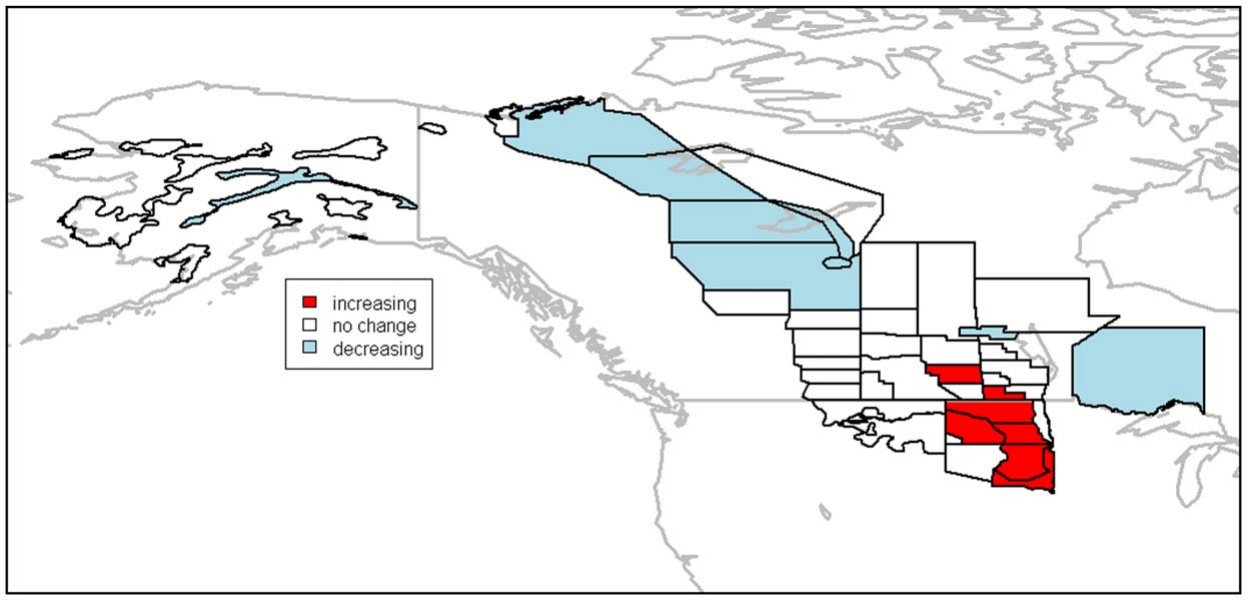
Increases and decreases in the abundance of breeding pairs since 1957 based on posterior distributions for the 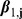 coefficients. Highlighted areas show an 95% chance of the population increasing (red) or decreasing (blue) in the area since 1957.

**Figure 4:**
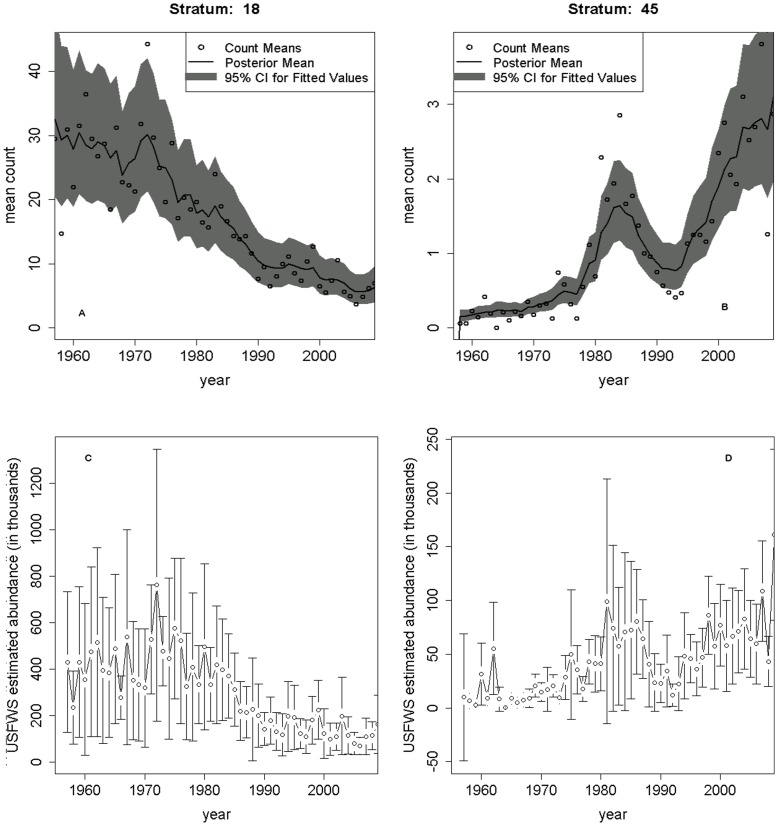
Marginal posterior predictive distributions from Strata 18 (A) and 45 (B) from the zero-inflated negative binomial model, and estimates of total scaup abundance from USFWS (C and D). Estimates from USFWS are calculated using counts of all scaup from aerial surveys adjusted by a visibility correction factor.

Mean point estimates from the ZINB model indicated that several strata in the boreal forest habitat had negative slope estimates, indicating a decrease in breeding pairs over time, while some of the strata in the prairie parkland habitat had positive slope estimates, indicating an increase in this region (Figs. 1 & 2). However, the majority (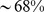) of the strata did not have significant increasing or decreasing trends in the abundance of paired scaup (*i.e.*, 95% credible intervals for the 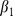 parameters overlapped zero; Fig. 2, Table S1). There were nevertheless several strata that experienced significant changes in population abundance across key areas of the scaup breeding range over the temporal extent of our study. When viewing each of these strata spatially, the northwest boreal forest of Canada and the southeastern prairie parkland region (PPR) both experienced significant changes in the abundance of paired scaup (when based on 95% credible intervals; Fig. 3). Time series of population abundance within each stratum can be examined individually to observe the combined temporal and spatial effects on breeding pairs. For example, apart from an abbreviated spike in the 1970s, the estimated time trend for stratum 18 in Canada’s northwest boreal forest indicated a steady decrease in the number of breeding pairs since the 1950s (Fig. 4a). When looking at the trend for stratum 45 in the prairies of North Dakota, the population increased up to the mid-1980s, decreased from the mid-1980s to the mid-1990s, and then increased dramatically since the mid-1990s (Fig. 4b). The zero-probability parameter associated with the ZINB model was estimated as 0.0204 (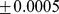). Parameter estimates for the spatial (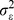) and temporal 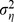 random effects were 81.41 (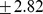) and 12.74 (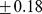), respectively, while that for the first-order autocorrelation in the temporal random effect was 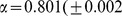).

## Discussion

### Scaup Decline

Our results provide further insight into the continental decline in scaup abundance. Due to uncertainty associated with the slope parameters, however, the majority of strata in the survey region did not indicate evidence of either a decline or increased breeding pair abundance (Fig. 2). This could imply that those strata in which there is a statistically significant decline in abundance are experiencing an intense decline, which appears to be the case for the northwestern boreal forest (Fig. 3), especially Strata 18 and 20 (Fig. 1). There were only 10 strata for which 95% credible intervals indicated significant population decline. It may be difficult to detect changes in breeding pair abundance, given the amount of sampling variation and autocorrelation present in the data (or the power to detect a trend is high, but it does not exist; this latter interpretation would be difficult to distinguish using the BPS data alone). Additionally, an examination of the individual strata indicates that the strata experiencing statistically significant increases in breeding pairs are experiencing dramatic increases, but initially had lower abundance (see Fig. 4).

Given these results, there are several possible processes underlying the observed changes in the abundance of paired scaup. Scaup may be choosing to forego migration up to the boreal forest, and are instead utilizing the PPR, where we found scaup to be increasing in abundance, at higher rates than in the past. Female scaup are however philopatric with>67% returning to their previous breeding site [53], [54], and a wholesale change in use of eco-regions does not seem likely. Rather, the birth-death balance has likely been negative for quite some time in the northwestern boreal forest, and positive in the southern PPR, perhaps due to the Conservation Reserve Program and other landscape management efforts that have increased nest success in this region [55]. While our results and others seem to indicate an increase in scaup numbers in the southern PPR [28], this increase is not large enough to offset the large decrease in the boreal forest (Fig. 1). While abundance in the southeastern PPR seems to have been growing exponentially since the mid-1990s, it is worth noting that the increments for the mean count of paired birds are quite small, ranging from only 1 to 3 (Fig. 1). This small growth does not offset the large decrease observed in the boreal forest (*e.g.*, Stratum 18, Fig. 4a). Recent studies indicate that current and future climate change may negatively impact late-nesting ducks such as scaup in the boreal forest and monitoring to assess these predicted declines is critical [41]. While these strata are only an example from each of the respective habitat types, there are only a few strata showing statistically significant increases and decreases that should be of most concern.

Our modeling approach differs substantially from the current methods used to estimate continental abundance of duck species [25], [56](Fig. 4). Specifically, the USFWS does not base management decisions on stratum-specific estimates and trends, but rather looks at how aggregated population size is changing at the continental scale. While the continental count can be related back to changes occurring in each stratum, an approach focused on trends in specific spatial regions may be more useful for guiding future management actions on the ground. Understanding why scaup are declining in certain areas of the boreal forest, and increasing in areas of the PPR is likely critical to preservation of scaup as a game species. It seems that the decrease in abundance of paired scaup is occurring on a much more local scale than previously thought, and restricting inference to the continental scale could inhibit identification of the mechanisms driving trends in key areas [28]. Furthermore, intensive monitoring of individual strata might be useful for management purposes, as detailed information on a finer spatial scale could help elucidate the causes and prevalence of population decline.

Our results may differ somewhat from similar studies [28] because of the time period taken into consideration, the focus on breeding-pair abundance, and the use of hierarchical models to separate observation error from process variation. If a different time period were used (*e.g.*, from 1955 to 1997 or 1978 to 1997, as in Afton and Anderson [28]), different conclusions may have been reached. Methodological changes occurred in 1975 that made it difficult to compare counts of grouped birds, and thus the total number of birds, from time periods before and after 1975. Our focus on breeding pairs alleviated this problem and allowed for a cohesive analysis from 1957 onward to identify spatially-explicit trends in abundance [57]. Additionally, our analysis was based on raw data of the segment-level counts of breeding pairs, rather than extrapolated estimates. The discrepancy between the counts and estimated abundance is notable, with raw counts in a stratum typically being less than 100 breeding pairs in a given year, and extrapolated estimates ranging up to hundreds of thousands of scaup (Fig. 4).

### Integrated Nested Laplace Approximation

Using INLA, we were able to successfully implement and compare several different models accounting for first- and second-order variation in the data across space and time, making this a more appropriate analysis of the BPS data. Although we could have used traditional MCMC methods, the processing time using INLA was considerably shorter, allowing us to fit models that might have otherwise not been considered due to computing limitations. For example, the processing time for the full negative binomial model was approximately 27 minutes on a 2 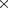 2.93 GHz 6-Core Intel Xeon workstation, making it roughly an order of magnitude faster than MCMC for this model and dataset (53 years of data with over 2500 segments sampled each year). While INLA does restrict the user to generalized linear models, it could be quite useful as an initial step for determining variables of importance to incorporate into a non-linear model using MCMC.

### Conclusions

Overall, our results support previous work indicating a decline in population abundance in the northern boreal forest of Canada, and additionally indicate that the population of scaup has increased rapidly in the southeastern PPR since 1957. Additionally, it seems that the most important processes influencing population dynamics were not related to second-order autocorrelation across strata or over time, but rather parameters in the model explicitly accounting for the differences among the strata (*i.e.*, the fixed intercept and slope parameters). Much of the variation in the data was explained by the fixed effects, and the temporal trend in the model was of greater importance in predicting dynamics than the random effects. This is not to say that the random effects were not important in our model, as properly accounting for autocorrelation can prevent erroneous conclusions. Our example could easily be further developed to accommodate covariates that might help explain underlying drivers of observed spatio-temporal variation in the population dynamics, such as information on environmental conditions (*e.g.*, drought conditions), predation, intensity of hunting, and other variables, 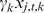, that could be used in place of the trend variable in (2). Further, additional measurement-level covariates (*e.g.*,observer) could be incorporated in (1) by letting the zero-inflation probability, 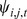, vary according to these other covariates. For example, if there were a single observation covariate, the model for 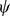 could be written as 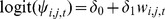 (or more flexibly by letting 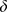 vary with *i,j,t* as well). If covariates were used to replace the trend parameters in the process model, the random effects would likely account for a greater amount of variation in the data relative to stratum-specific trends. Spatially-explicit effects of intra- and inter-specific density dependence could also be included in the INLA framework using the log-linear parameterizations of the Gompertz or Ricker models [3], [6]. These could simply be implemented as linear covariates along with environmental covariates [12]. Additionally, results from the INLA analysis can be used to better inform the way in which the BPS is conducted, using an optimal sampling scheme to maximize the amount of information gathered during the survey while minimizing associated error [58].

## Supporting Information

Text S1 R code for INLA and related analysis.
(DOC)

Table S1Estimates of the mean, standard deviation, and 95% credible interval for the 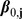 and 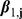 parameters from the zero-inflated negative binomial model with random spatio-temporal effects.(DOCX)
