# Supplementary material for: An Accessible Method for Implementing Hierarchical Models with Spatio-Temporal Abundance Data
Source: PLoS One. 2012 Nov 16;7(11):e49395. doi: 10.1371/journal.pone.0049395 (PMC3500297; doi:10.1371/journal.pone.0049395)
Supplement: Text S1 — R code for INLA and related analysis. (DOC) [file pone.0049395.s001.doc]

Text S1

R code for INLA and related analysis

Analysis conducted using R version 2.12

#### Load Libraries

library(INLA)

#### Prepare Model 1 Formula for use with INLA

#### This given formula involves covariates (`trend'), as well #### as a spatial field, given in the file `W.dat', and a term #### for temporal autocorrelation given by the "model='ar1'" #### command

formula.tmp=paste(trend,"+f(stratum,model='besag',

graph.file='W.dat',replicate=year)+f(year,model='ar1',replicate=stratum)",sep="")

formula.1=as.formula(formula.tmp)

#### Fit ICAR NegBinom and ZINB{1} Trend Model

#### Here, the inla command needs the formula from the previous #### step, as well as specification of the likelihood (`family') #### and the data.

#### For our example, the 'E' in the formula is a known constant #### (the number of segments in each stratum in a given year) #### and acts like an offset in the model.

inla.negbinom=inla(formula.1,family="nbinomial",E=N,data=counts.sum.reg.df)

inla.zinb=inla(formula.1,family="zeroinflatednbinomial1",E=N,

data=counts.sum.reg.df)
