# Supplementary material for: An Accessible Method for Implementing Hierarchical Models with Spatio-Temporal Abundance Data
Source: PLoS One. 2012 Nov 16;7(11):e49395. doi: 10.1371/journal.pone.0049395 (PMC3500297; doi:10.1371/journal.pone.0049395)
Supplement: Table S1 — Estimates of the mean, standard deviation, and 95% credible interval for the and parameters from the zero-inflated negative binomial model with random spatio-temporal effects. (DOCX) [file pone.0049395.s002.docx]

Table S2: Estimates of the mean, standard deviation (sd), and 95% credible interval for the β_0,_*_j_* and β_1,_*_j_* parameters from the zero-inflated negative binomial model with random spatio-temporal effects. The *j* subscripts refer to the stratum number.

| parameter | mean | sd | 0.025  quantile | 0.975  quantile |
| --- | --- | --- | --- | --- |
| β_0,1_ | -0.3336 | 0.2636 | -0.8506 | 0.1831 |
| β_0,2_ | 1.5238 | 0.2394 | 1.0551 | 1.9943 |
| β_0,3_ | 2.2578 | 0.2370 | 1.7938 | 2.7236 |
| β_0,4_ | 2.8016 | 0.2382 | 2.3353 | 3.2698 |
| β_0,5_ | 1.4371 | 0.2440 | 0.9593 | 1.9166 |
| β_0,6_ | 1.6488 | 0.2380 | 1.1829 | 2.1164 |
| β_0,7_ | 2.1754 | 0.3161 | 1.5591 | 2.7994 |
| β_0,8_ | 1.4899 | 0.2396 | 1.0208 | 1.9606 |
| β_0,9_ | 1.8271 | 0.2363 | 1.3645 | 2.2915 |
| β_0,10_ | 1.9545 | 0.2393 | 1.4859 | 2.4247 |
| β_0,11_ | 2.0039 | 0.2374 | 1.5392 | 2.4703 |
| β_0,12_ | 3.0406 | 0.2386 | 2.5735 | 3.5097 |
| β_0,13_ | 2.4869 | 0.2355 | 2.0259 | 2.9498 |
| β_0,14_ | 2.2936 | 0.2334 | 1.8367 | 2.7525 |
| β_0,15_ | 1.7724 | 0.2356 | 1.3112 | 2.2354 |
| β_0,16_ | 1.4509 | 0.2341 | 0.9928 | 1.9110 |
| β_0,17_ | 1.6353 | 0.2338 | 1.1777 | 2.0948 |
| β_0,18_ | 3.6235 | 0.2337 | 3.1662 | 4.0830 |
| β_0,20_ | 2.8620 | 0.2358 | 2.4006 | 3.3256 |
| β_0,21_ | -0.0210 | 0.2395 | -0.4901 | 0.4495 |
| β_0,22_ | 1.1117 | 0.2342 | 0.6533 | 1.5722 |
| β_0,23_ | 0.0440 | 0.2319 | -0.4098 | 0.4997 |
| β_0,24_ | -0.0192 | 0.2325 | -0.4743 | 0.4377 |
| β_0,25_ | 2.2542 | 0.2313 | 1.8014 | 2.7090 |
| β_0,26_ | 2.1789 | 0.2315 | 1.7259 | 2.6339 |
| β_0,27_ | 1.1166 | 0.2292 | 0.6679 | 1.5670 |
| β_0,28_ | 0.8683 | 0.2306 | 0.4169 | 1.3216 |
| β_0,29_ | 0.4288 | 0.2331 | -0.0276 | 0.8869 |
| β_0,30_ | 0.9017 | 0.2301 | 0.4513 | 1.3540 |
| β_0,31_ | 0.5432 | 0.2317 | 0.0896 | 0.9987 |
| β_0,32_ | -0.0174 | 0.2299 | -0.4673 | 0.4345 |
| β_0,33_ | -0.7787 | 0.2497 | -1.2682 | -0.2886 |
| β_0,34_ | 0.0909 | 0.2312 | -0.3616 | 0.5451 |
| β_0,35_ | 0.3714 | 0.2344 | -0.0875 | 0.8321 |
| β_0,36_ | -0.5852 | 0.2946 | -1.1651 | -0.0095 |
| β_0,37_ | 0.6653 | 0.2345 | 0.2063 | 1.1260 |
| β_0,38_ | -2.4785 | 0.3702 | -3.2203 | -1.7671 |
| β_0,39_ | 0.2642 | 0.2342 | -0.1943 | 0.7245 |
| β_0,40_ | 1.5929 | 0.2312 | 1.1403 | 2.0474 |
| β_0,41_ | -0.8200 | 0.3169 | -1.4403 | -0.1973 |
| β_0,42_ | -2.1544 | 0.3397 | -2.8204 | -1.4879 |
| β_0,43_ | -2.7003 | 0.3409 | -3.3750 | -2.0379 |
| β_0,44_ | -2.0511 | 0.3199 | -2.6756 | -1.4205 |
| β_0,45_ | -1.9319 | 0.2581 | -2.4379 | -1.4256 |
| β_0,46_ | -1.8632 | 0.2622 | -2.3776 | -1.3493 |
| β_0,47_ | -4.1585 | 0.9988 | -6.3004 | -2.3774 |
| β_0,48_ | -1.8723 | 0.2629 | -2.3872 | -1.3561 |
| β_0,49_ | -1.9172 | 0.3086 | -2.5254 | -1.3151 |
| β_0,50_ | -0.7990 | 0.2605 | -1.3096 | -0.2877 |
| β_0,75_ | 1.5700 | 0.2353 | 1.1094 | 2.0326 |
| β_0,76_ | 1.1170 | 0.2328 | 0.6612 | 1.5744 |
| β_0,77_ | 0.9219 | 0.2333 | 0.4652 | 1.3805 |
| β_1,1_ | 0.0063 | 0.0083 | -0.0099 | 0.0225 |
| β_1,2_ | 0.0054 | 0.0075 | -0.0094 | 0.0202 |
| β_1,3_ | -0.0157 | 0.0075 | -0.0304 | -0.0011 |
| β_1,4_ | -0.0064 | 0.0075 | -0.0211 | 0.0084 |
| β_1,5_ | -0.0087 | 0.0077 | -0.0239 | 0.0065 |
| β_1,6_ | -0.0061 | 0.0075 | -0.0208 | 0.0086 |
| β_1,7_ | -0.0380 | 0.0089 | -0.0555 | -0.0206 |
| β_1,8_ | 0.0072 | 0.0076 | -0.0076 | 0.0220 |
| β_1,9_ | 0.0052 | 0.0074 | -0.0094 | 0.0198 |
| β_1,10_ | 0.0000 | 0.0075 | -0.0148 | 0.0148 |
| β_1,11_ | 0.0035 | 0.0075 | -0.0111 | 0.0182 |
| β_1,12_ | 0.0017 | 0.0075 | -0.0130 | 0.0164 |
| β_1,13_ | -0.0120 | 0.0074 | -0.0265 | 0.0026 |
| β_1,14_ | -0.0208 | 0.0074 | -0.0352 | -0.0064 |
| β_1,15_ | -0.0229 | 0.0074 | -0.0375 | -0.0084 |
| β_1,16_ | -0.0143 | 0.0074 | -0.0289 | 0.0002 |
| β_1,17_ | -0.0198 | 0.0073 | -0.0342 | -0.0055 |
| β_1,18_ | -0.0343 | 0.0074 | -0.0488 | -0.0198 |
| β_1,20_ | -0.0365 | 0.0075 | -0.0512 | -0.0217 |
| β_1,21_ | 0.0013 | 0.0074 | -0.0132 | 0.0158 |
| β_1,22_ | -0.0136 | 0.0074 | -0.0280 | 0.0009 |
| β_1,23_ | -0.0089 | 0.0073 | -0.0231 | 0.0054 |
| β_1,24_ | -0.0063 | 0.0073 | -0.0206 | 0.0079 |
| β_1,25_ | -0.0198 | 0.0073 | -0.0340 | -0.0056 |
| β_1,26_ | -0.0118 | 0.0073 | -0.0261 | 0.0025 |
| β_1,27_ | -0.0126 | 0.0072 | -0.0268 | 0.0016 |
| β_1,28_ | -0.0044 | 0.0073 | -0.0187 | 0.0099 |
| β_1,29_ | -0.0039 | 0.0074 | -0.0183 | 0.0106 |
| β_1,30_ | 0.0059 | 0.0072 | -0.0083 | 0.0200 |
| β_1,31_ | -0.0015 | 0.0073 | -0.0158 | 0.0128 |
| β_1,32_ | 0.0044 | 0.0073 | -0.0099 | 0.0186 |
| β_1,33_ | -0.0021 | 0.0079 | -0.0176 | 0.0134 |
| β_1,34_ | 0.0149 | 0.0073 | 0.0006 | 0.0291 |
| β_1,35_ | -0.0058 | 0.0074 | -0.0204 | 0.0088 |
| β_1,36_ | 0.0062 | 0.0090 | -0.0113 | 0.0239 |
| β_1,37_ | -0.0092 | 0.0074 | -0.0236 | 0.0052 |
| β_1,38_ | 0.0018 | 0.0119 | -0.0215 | 0.0251 |
| β_1,39_ | 0.0171 | 0.0074 | 0.0027 | 0.0316 |
| β_1,40_ | -0.0064 | 0.0073 | -0.0207 | 0.0079 |
| β_1,41_ | 0.0052 | 0.0092 | -0.0129 | 0.0233 |
| β_1,42_ | 0.0052 | 0.0099 | -0.0143 | 0.0247 |
| β_1,43_ | 0.0409 | 0.0098 | 0.0218 | 0.0603 |
| β_1,44_ | 0.0073 | 0.0097 | -0.0119 | 0.0263 |
| β_1,45_ | 0.0586 | 0.0079 | 0.0432 | 0.0741 |
| β_1,46_ | 0.0637 | 0.0080 | 0.0481 | 0.0794 |
| β_1,47_ | -0.0051 | 0.0310 | -0.0657 | 0.0559 |
| β_1,48_ | 0.0407 | 0.0080 | 0.0249 | 0.0565 |
| β_1,49_ | 0.0212 | 0.0092 | 0.0033 | 0.0393 |
| β_1,50_ | -0.0400 | 0.0094 | -0.0582 | -0.0212 |
| β_1,75_ | -0.0042 | 0.0074 | -0.0187 | 0.0104 |
| β_1,76_ | -0.0135 | 0.0073 | -0.0278 | 0.0007 |
| β_1,77_ | -0.0240 | 0.0073 | -0.0384 | -0.0096 |
